# Diagnostic performance of ct angiography for gastrointestinal haemorrhage according to the clinical severity

**DOI:** 10.1186/2197-425X-3-S1-A605

**Published:** 2015-10-01

**Authors:** YJ Choi, KS Kim, GJ Suh, WY Kwon, KM Yoo, JS Kim

**Affiliations:** Department of Emergency Medicine, Seoul National University Hospital, Seoul, Republic of Korea

## Introduction

Acute gastrointestinal haemorrhage (GIH) is a common medical emergency with a significant morbidity and mortality. Although endoscopy is considered as a primary diagnostic modality, computed tomography (CT) angiography is recently introduced as an alternative diagnostic tool because of its advantages such as readiness, wide anatomic coverage, and minimal invasiveness. However, no studies have evaluated the diagnostic performance of CT angiography according to the clinical severity of GIH.

## Objectives

The objective of this study was to compare the diagnostic performance of CT angiography in patients with the different severity of GIH.

## Methods

This study was performed in single tertiary teaching hospital ED. We have retrospectively identified all adult patients who have received GIH protocol CT angiography during 2 years. Patients with trauma or without further diagnostic workup were excluded. The results of CT angiography was considered as positive if there was any signs of active extravasations of contrast materials, enhancement of the bowel wall, presence of vascular abnormalities, polyp, or tumor. The reference standard consisted of esophagogastroduodenoscopy, colonoscopy, sigmoidoscopy, angiography, bleeding scan, capsule endoscopy and surgery, either alone or in combination. Clinical severity was stratified according to the amount of transfused RBC during first 2 days. Patients were categorized into mild (1st quartile), moderate (2nd and 3rd quartile), and severe group (4th quartile). Diagnostic performance was measure by sensitivity, specificity, area under receiver operating characteristics curve (AUC), positive predictive value (PPV), and negative predictive value (NPV).

## Results

**Figure 1:**
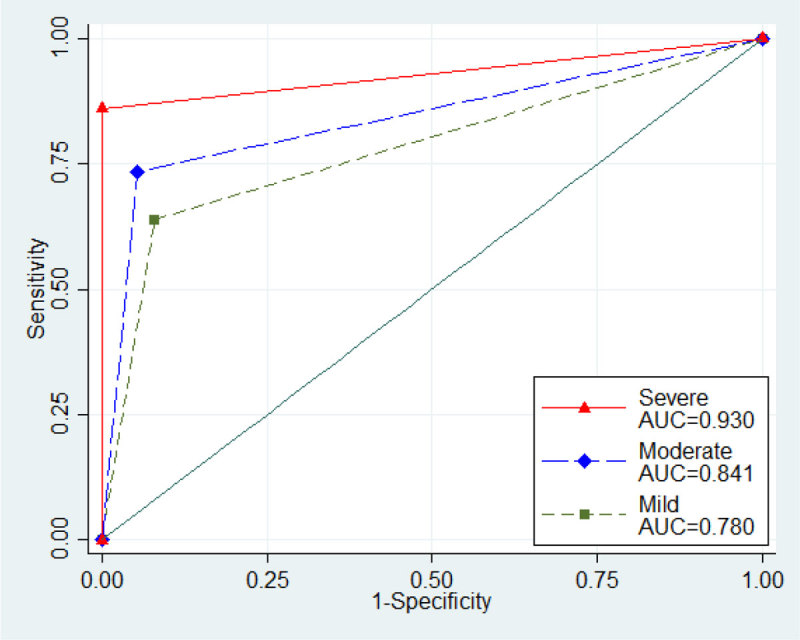
Figure 1

Among 262 cases analyzed, 75 (28.6%), 139 (53.1%), and 48 (18.3%) were categorized as mild, moderate and severe group, respectively. Severe group was more likely to have hematemesis as presenting symptoms. And severe group had lower blood pressure and lower hemoglobin level. Patients with severe GIH had a tendency to receive conventional angiography more frequently (mild [9/75, 12%], moderate [42/139, 30.2%], and severe [28/48, 58.3%]). Diagnostic performance of CT angiography was significantly higher in more severe GIH (Table 1 and Figure 1).

**Table 1:** Diagnostic performance of CT angiography

	Prevalence	Sensitivity	Specificity	AUC	PPV	NPV
All patients (N = 262)	74.4 (68.7-79.6)	73.8 (67.1-79.6)	94.0 (85.4-98.3)	.839 (.797-.881)	97.3 (93.2-99.3)	55.3 (45.7-64.6)
Mild (n = 75)	66.7 (54.8-77.1)	64.0 (49.2-77.1)	92.0 (74.0-99.0)	.780 (.694-.866)	94.1 (80.3-99.3)	56.1 (39.7-71.5)
Moderate (n = 139)	73.4 (65.2-80.5)	73.5 (63.9-81.8)	94.6 (81.8-99.3)	.841 (.784-.897)	97.4 (90.9-99.7)	56.5 (43.3-69.0)
Severe (n = 48)	89.6 (77.3-96.5)	86.0 (72.1-94.7)	100 (47.8-100)	.930 (.878-.983)	100 (90.5-100)	45.5 (16.7-76.6)
Data were described as percentages with 95% confidence intervals except AUC. AUC, area under the receiver operating characteristics curve; PPV, positive predictive value; NPV, negative predictive value.

## Conclusions

Diagnostic performance of CT angiography is better in patients with more severe GIH.
